# Paeoniflorin Attenuates Limb Ischemia by Promoting Angiogenesis Through ERα/ROCK-2 Pathway

**DOI:** 10.3390/ph18020272

**Published:** 2025-02-19

**Authors:** Mengyao Li, Qianyi Wang, Sinan Zhu, Wei Sun, Xiuyun Ren, Zhenkun Xu, Xinze Li, Shaoxia Wang, Qi Liu, Lu Chen, Hong Wang

**Affiliations:** 1 School of Medical Technology, Tianjin University of Traditional Chinese Medicine, Tianjin 301600, China; limyao2020@163.com (M.L.); wend13193586506@163.com (Q.W.); liuqi23@tjutcm.edu.cn (Q.L.); 2 Instrumental Analysis and Research Center, Tianjin University of Traditional Chinese Medicine, Tianjin 301600, China; 3 School of Public Health and Health Sciences, Tianjin University of Traditional Chinese Medicine, Tianjin 301600, China

**Keywords:** paeoniflorin, hind limb ischemia, angiogenesis, endothelial cells, inflammation, ERα/ROCK-2 pathway

## Abstract

**Background:** Peripheral artery disease (PAD) is a high-risk vascular condition, and vascular remodeling has become a promising therapeutic approach. Paeoniflorin (PF) is the main bioactive compound in the roots of *Paeonia lactiflora* Pall, which is commonly used to treat a range of cardiovascular disorders. However, the mechanisms underlying the ameliorating effects of PF on PAD remain unclear. Therefore, the purpose of this study was to explore the therapeutic efficiency of PF on PAD and determine its mechanisms. **Methods:** The blood flow of mice was detected with a laser Doppler dot scanning imaging system. HE staining was used to observe the morphological changes of ischemic muscle. The changes in the serologic indexes were detected with an automatic biochemical assay, and the capillary density of ischemic gastrocnemius was detected with a Lectin immunofluorescence assay. The expression of angiogenesis-related proteins in ischemic gastrocnemius was detected with Western blotting, and the proportion of macrophages and neutrophils in total cells was detected with flow cytometry. **Results:** PF significantly increased blood flow, capillary density and protein expressions of vascular endothelial growth factor A (VEGFA), matrix metalloproteinase 2 (MMP2), matrix metalloproteinase 2 (MMP9), and estrogen receptor α (ERα) in mouse ischemic tissue in a PAD model. PF enhances the migration of endothelial cells and promotes the formation of tubular structures, involving the ERα/ROCK2 signaling pathway. Furthermore, PF was found to promote the phenotypic transformation of macrophages and alleviated grave inflammatory responses during vascular remodeling. **Conclusions:** We determined that PF as a potent compound in promoting angiogenesis and mitigating inflammatory responses during revascularization.

## 1. Introduction

Peripheral artery disease (PAD) is a hallmark of systemic atherosclerosis and represents a prevalent and significant public health issue. Epidemiological data indicate that PAD has a global prevalence of 5.8%, affecting approximately 237 million people worldwide [1,2]. Currently, therapeutic strategies for PAD primarily prioritize pharmacotherapy and interventional and surgical approaches [3]. However, treatment outcomes are ultimately unsatisfactory due to the influence of comorbidities, disease severity, and the effectiveness of timely interventions. Consequently, there is a pressing need to develop more effective treatments for PAD.

Angiogenesis, a fundamental physiological and pathological process, is considered essential for treating lesions in vital organs and tissues [4]. In the presence of tissue ischemia, angiogenesis improves blood circulation and restores blood supply to local tissues, facilitating tissue recovery from ischemic lesions. Thus, angiogenesis is regarded as a promising therapeutic strategy and a cornerstone for limb preservation in PAD patients.

Angiogenesis and inflammation exhibit strong interdependency [5,6]. Notably, angiogenesis is frequently accompanied by inflammation, thereby limiting the efficacy of therapeutic angiogenesis alone in achieving desired outcomes [7]. Excessive angiogenesis can maintain chronic inflammation [8,9]. Macrophage polarization plays an important role during inflammation [10]. Enhancing the levels of vascular endothelial growth factor (VEGF) facilitates earlier recruitment of a greater number of macrophages, leading to the subsequent transition of macrophage phenotypes from pro-inflammatory to anti-inflammatory [11]. Increased anti-inflammatory macrophages in the later stages of inflammation contribute to inflammation alleviation, the initiation of angiogenesis, and tissue repair enhancement. However, excessive pro-inflammatory macrophages exacerbate ischemia and delay recovery [12,13,14]. It is crucial to inhibit macrophage polarization while promoting angiogenesis for effective PAD treatment.

*Paeonia lactiflora* Pall, commonly referred to as “Shao Yao” in China, is a popular herbal medicine used to activate blood circulation and remove blood stasis [15]. Paeoniflorin (PF), a key bioactive compound in *Paeonia lactiflora* roots, shows notable anti-inflammatory, antioxidant, and immune-modulatory properties [16,17,18]. Emerging evidence also highlights PF’s roles in antithrombotic effects and angiogenesis [17,19,20]. In the study, we evaluated PF’s effectiveness in treating PAD in vivo and elucidated its mechanism of action in promoting angiogenesis and inhibiting inflammation in vitro. Our findings support the use of PF for treating ischemic diseases.

## 2. Results

### 2.1. PF Improved Hindlimb Ischemia in C57BL/6 Mice

**Figure 1:**
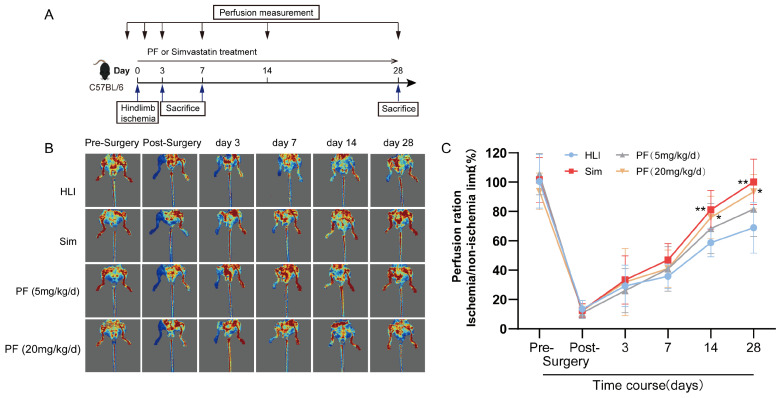
PF improved hindlimb ischemia in HLI mice. (**A**) The scheme of the experimental setup was presented. (**B**) Illustrative blood perfusion images at various time points (pre-surgery and post-surgery and 3, 7, 14, and 28 days following femoral artery removal) were shown for the four groups. Blue areas indicate regions with severe ischemia, red areas indicate regions without ischemia or with mild ischemia. (**C**) Blood perfusion was quantified at various time points. Data points are displayed as mean ± SD, n = 6. * *p* < 0.05, ** *p* < 0.01 vs. HLI.

To investigate the PF’s potential effects on ischemic injury, we performed a hindlimb ischemia operation on C57BL/6 mice following the experimental scheme illustrated in Figure 1A. The results revealed that PF did not significantly impact body weight or gastrocnemius muscle weight in the HLI models (Supplementary Figure S1A,B). Moreover, continuous administration of PF for 28 days did not affect liver and kidney function in the mice (Supplementary Figure S1C–I). As presented in Figure 1B,C, in comparison to the HLI model group, representative images of blood flow perfusion showed distinct improvements in blood flow in both the sim group and the PF-treated groups with low and high doses. These findings suggested that PF enhances perfusion recovery in mice with hindlimb ischemia.

### 2.2. PF Reduced Tissue Injury in HLI Mouse

**Figure 2:**
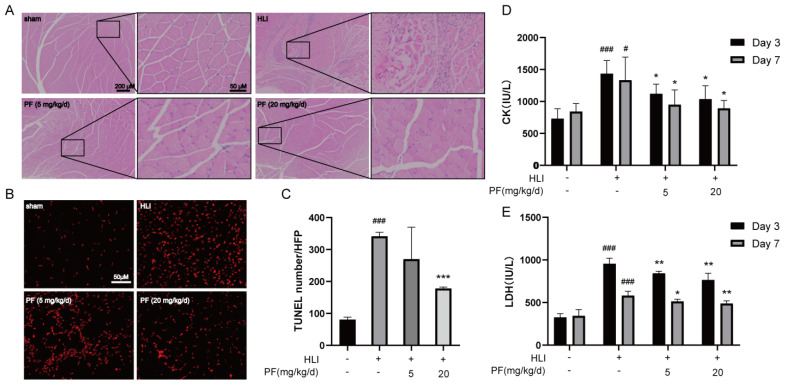
PF promoted the repair of ischemic gastrocnemius injury in an HLI mouse model. (**A**) H&E-stained muscle tissue in the hindlimb at 28 days after surgery were presented. The scale bar indicates 200 μm (left) and 50 μm (right). (**B**,**C**) TUNEL staining detected apoptosis (red) in the gastrocnemius at 7 days after surgery. The scale bar indicates 50 μm. The data points are represented as mean ± SD, n = 3. (**D**) Serum CK levels in mice were measured at 3 and 7 days after surgery. (**E**) Serum LDH levels in mice were measured at 3 and 7 days after surgery. The data points are represented as mean ± SD, n = 4. ^#^ *p* < 0.05, ^###^ *p* < 0.001 vs. Sham; * *p* < 0.05, ** *p* < 0.01, *** *p* < 0.001 vs. HLI.

H&E staining was utilized to detect histopathological features of the gastrocnemius at day 28 after operation. Histologic assessment revealed that mice treated with PF exhibited more regular tissue morphology in the ischemic gastrocnemius and smaller gaps among muscle bundles compared to the HLI group, indicating that PF significantly reduced tissue injury in the ischemic gastrocnemius (Figure 2A). A TUNEL assay was performed on gastrocnemius sections for studying apoptosis at day 7 after operation (Figure 2B). Compared to the HLI group, the PF group had a dramatic decline in the count of TUNEL-stained positive signals in the ischemic tissue, indicating that PF significantly decreased apoptosis in the gastrocnemius tissue of HLI mice (Figure 2C). Moreover, the serum results demonstrated that CK and LDH levels visibly increased in the HLI group but were prevented by PF administration (Figure 2D,E). In summary, these findings indicated that PF promotes tissue injury repair in mice with HLI.

### 2.3. PF Promoted Angiogenesis in Gastrocnemius Muscle

**Figure 3:**
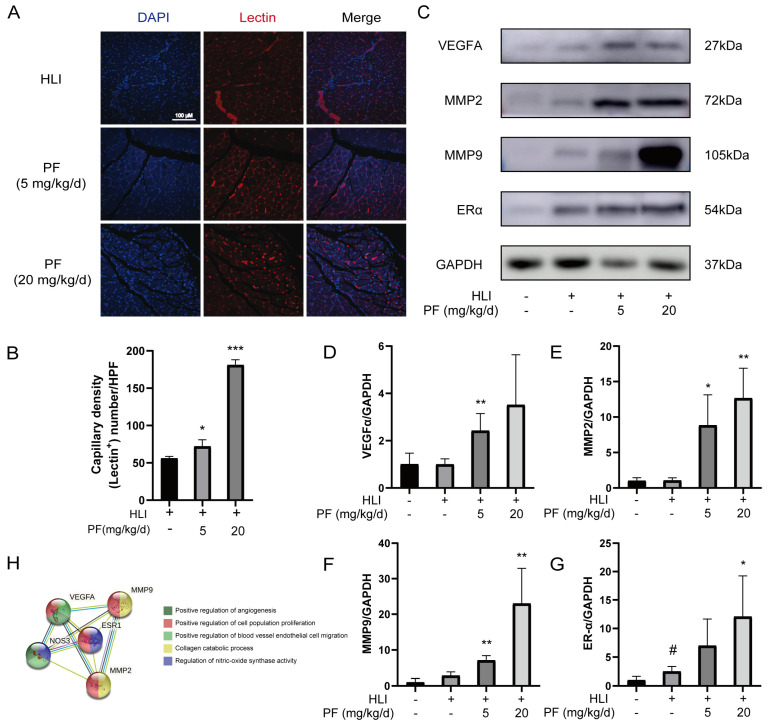
PF-promoted angiogenesis of ischemic gastrocnemius in an HLI mouse model. (**A**) Representative immunofluorescent images illustrate capillary density in the ischemic muscles of mice 28 days post-surgery, with or without PF treatment at low or high doses. Lectin (red) was utilized to label capillaries, and DAPI (blue) was applied for nuclei. Scale bar: 100 μm. (**B**) Quantification of capillary density is expressed as lectin-positive number per randomly chosen high-power field (HPF). The data points are represented as mean ± SD, n = 3. (**C**) The Western blot analysis of VEGFA, MMP2, MMP9, and ERα protein levels in gastrocnemius on post-surgery day 7. GAPDH was employed as a loading control. (**D**–**G**) The quantification of protein expression ratios of VEGFA, MMP2, MMP9, and ERα to GAPDH in gastrocnemius on post-surgery day 7. Data points are shown as mean ± SD, n = 3. (**H**) A STRING analysis was carried out for angiogenesis-related factors interaction. ^#^ *p* < 0.05 vs. Sham; ^*^ *p* < 0.05, ** *p* < 0.01, *** *p* < 0.001 vs. HLI.

The increase in capillary density positively contributes to blood flow recovery in ischemic tissues [21,22]. The capillary density of mice was measured by lectin immunofluorescence staining at 28 days after operation. Positive lectin signals, representing capillaries, were notably higher in the PF group, suggesting a dramatic increase in capillary density in the gastrocnemius of HLI mice treated with PF (Figure 3A,B). In addition, Western blotting was used to measure angiogenesis-related proteins in mice ischemic gastrocnemius tissue. The results revealed that PF increased VEGFA, MMP2, MMP9, and ERα protein expression at 7 days post-HLI compared to the HLI group (Figure 3C–G). The STRING analysis demonstrated interactions among these proteins (Figure 3H), a higher number of connecting lines indicates a stronger interaction between the proteins. This study demonstrates that PF promotes angiogenesis, mitigates ischemic tissue injury, and enhances endothelial cell function by modulating the protein expression of VEGFA, MMP2, MMP9, and ERα, leading to increased capillary density in ischemic tissues.

### 2.4. PF Promoted the Proliferation, Migration, and Tube Formation of HUVECs

**Figure 4:**
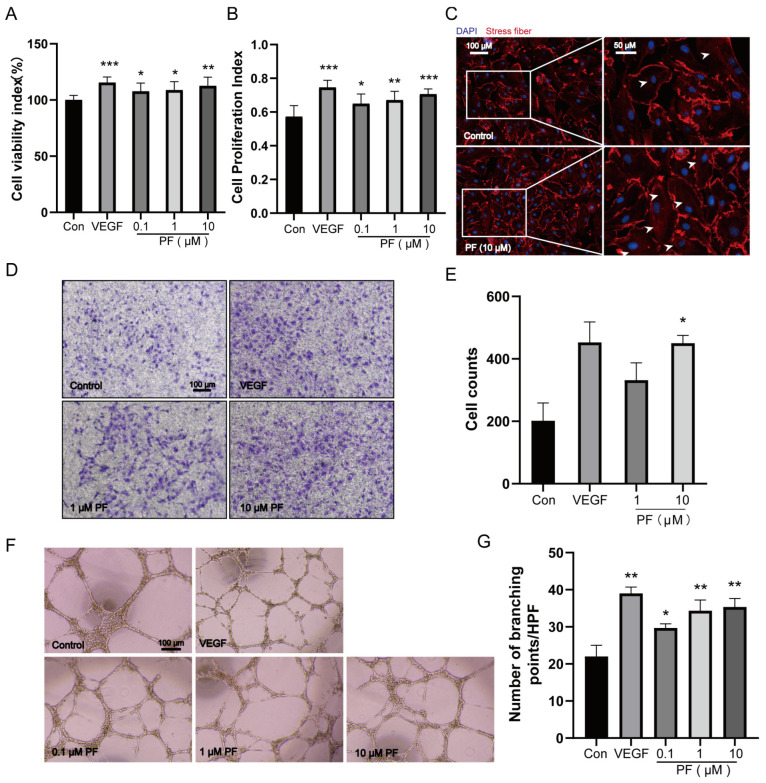
PF promoted the proliferation, migration, and tube formation of HUVECs. (**A**) The cell viability of HUVECs was assessed 24 h after PF administration using CCK8 assays (n = 6). (**B**) Brdu assays were performed to assess HUVECs’ cell proliferation for 24 h after the administration of PF (n = 7). (**C**) Representative images of actin cytoskeleton rearrangement in HUVECs were visible (n = 3). Stress fibers terminated at the tapered termini, as indicated by the white arrowheads. Scale bar = 100 μm (left), scale bar = 50 μm (right). (**D**) Transwell invasion assays of HUVECs treated with DMSO or PF (1 and 10 μM) were performed (n = 3). The scale bars indicated 100 μm. (**E**) Transwell quantification was expressed as cell counts (n = 3). (**F**) HUVEC tube formation on Matrigel was assessed in response to DMSO, PF (0.1, 1, and 10 μM), or VEGFA (100 ng/mL) for 18 h. The scale bars represented 100 μm. (**G**) The quantification of tube formation was presented as several branching points (n = 3). VEGF is a positive control; the data points are displayed as mean ± SD. * *p* < 0.05, ** *p* < 0.01, *** *p* < 0.001 vs. Control.

Endothelial cell proliferation and migration are essential for neovascularization [23]. To verify the consequence of PF on angiogenesis processes, we performed cell experiments, including angiogenesis assessments and vascular endothelial cell migration assays. Both PF and VEGF enhanced the viability and proliferation ability of HUVECs (Figure 4A,B). Additionally, the immunofluorescence analysis revealed that PF (10 μM) caused actin cytoskeleton rearrangement and the formation of actin stress fibers, which are crucial for endothelial cell migration (Figure 4C). We investigated the influence of PF on HUVEC migration through a Transwell assay, which showed a notable rise in the number of HUVECs traversing the chambers relative to the control group (Figure 4D,E). Notably, Matrigel tube formation assays demonstrated that both VEGF and PF (1, 10 μM) increased the number of vascular branch points and enhanced tube formation capacity of HUVECs compared to the control group (Figure 4F,G). These findings ascertained that PF promoted the proliferation, migration, and tube formation of HUVECs, suggesting its potential role in angiogenesis.

### 2.5. PF Induced HUVECs Migration and Tube Formation by Modulating ERα/ROCK-2 Signaling Pathway

**Figure 5:**
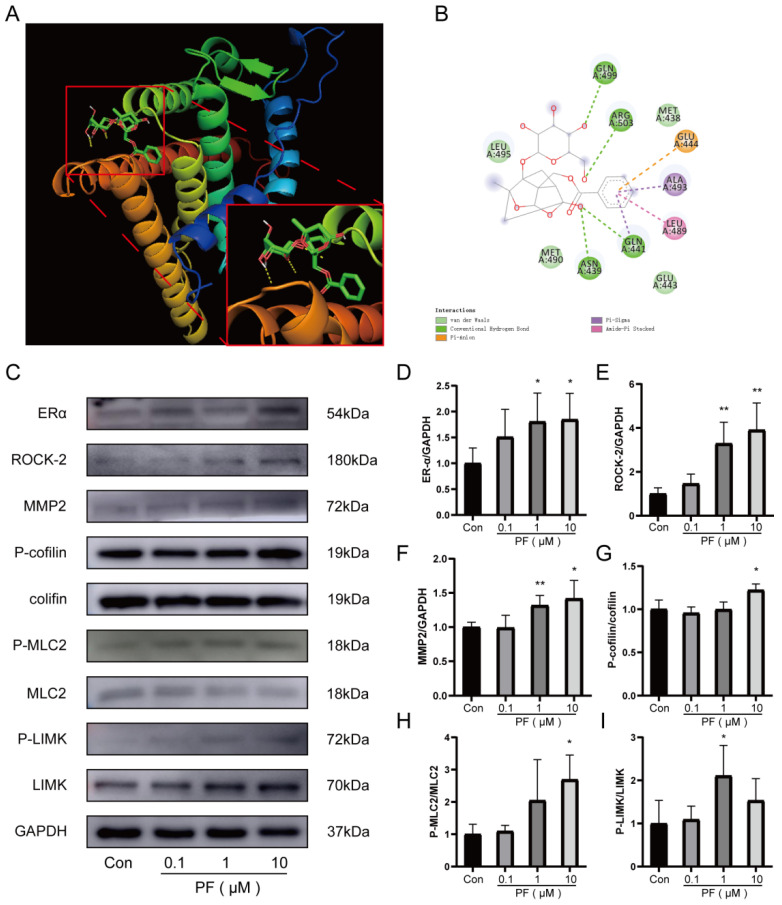
PF stimulated HUVECs’ migration and tube formation in HUVECs through the ERα/ROCK-2 pathway. (**A**,**B**) The diagram of the molecular docking structure and interaction of PF and ERα were performed. (**C**) The Western blot analysis of ERα, ROCK-2, P-LIMK, LIMK, P-cofilin, cofilin, P-MLC2, MLC2, and MMP2 protein expression in HUVECs treated with DMSO or PF (0.1, 1, and 10 μM) for 24 h. GAPDH acted as a loading control. (**D**–**I**) The quantification of protein levels of ERα, ROCK-2, and MMP2 to GAPDH and P-LIMK to LIMK, P-cofilin to cofilin, and P-MLC2 to MLC2 treated with DMSO or PF (0.1, 1 and 10 μM) in HUVECs for 24 h. The data points are represented as mean ± SD, n = 3. * *p* < 0.05, ** *p* < 0.01 vs. control.

To explore the mechanism underlying the enhancement of HUVEC tube formation capability mediated by PF, RT-PCR was utilized to assess the impact of PF on gene regulation associated with endothelial cell tube formation. PF was found to regulate the mRNA expression of tube formation-related genes, including ROCK-2, PROK2, PPARG, ERα, VEGFR (Supplementary Figure S2A–H), etc. In vivo, PF facilitated angiogenesis by upregulating the expression of ERα in ischemic gastrocnemius. A binding affinity assessment of PF towards ERα was conducted through a docking analysis. These results revealed that the binding energy obtained is −7.0 (kcal/mol) through the formation of intermolecular forces that include hydrogen bonds and van der Waals forces, etc., that indicated a desirable binding capability between PF and ERα (Figure 5A,B). According to previous research, the Rho GTPase-RhoA regulates the dynamics of the actin cytoskeleton and stress fiber formation of endothelial cells [24]. ERα activates the expression of its main downstream factor, known as Rho-associated protein kinase (ROCK-2), to enhance the contractility of endothelial cells through non-genomic action, sabotage the adhesion between cells, and boost endothelial cell migration, thereby affecting the tube formation capability of endothelial cells [25]. We tested the level of ROCK-2. The findings indicated PF elevated the expression of ROCK-2 protein. Then, we subsequently employed Western blotting to examine the levels of related proteins in the ROCK-2 pathway, MMP2, LIMK, MLC, and cofilin. Our experimental results manifested that PF remarkably upregulated the expression of MMP2 in HUVECs. Moreover, PF dramatically escalated the phosphorylation of LIMK, cofilin, and MLC2 in HUVECs (Figure 5C–I). The aforementioned results indicated that PF promoted the formation of endothelial stress fibers and escalated the capability of endothelial cells on tube formation in vitro through exerting the effect of endothelial cell migration by activating the ERα/ROCK-2 pathway.

### 2.6. PF Resolved Inflammation During HLI

**Figure 6:**
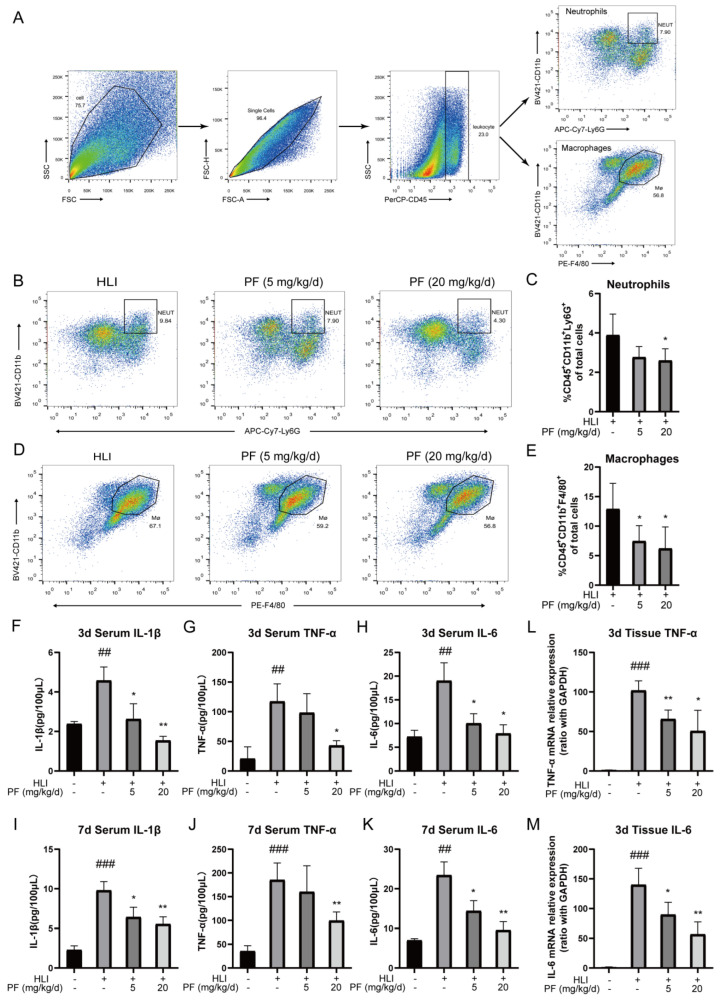
PF resolved inflammation during HLI. (**A**) The diagram of flow cytometric selection strategy of macrophages and neutrophils in HLI were shown. (**B**) Typical flow cytometry images of neutrophils in mice treated or not with PF (lower and high dose) on day 3 after surgery. Quantitative data were shown in (**C**) (n = 4–5). (**D**) Typical flow cytometry images of macrophages in mice treated or not with PF (lower and high dose) on day 3 after surgery. Quantitative data were shown in (**E**) (n = 4–5). (**F**–**K**) Serum concentrations of IL-1β, TNF-α, and IL-6 were quantified by an ELISA analysis on day 3 and day 7 after surgery (n = 3). (**L**–**M**) RT-PCR was utilized to examine TNF-α and IL-6 gene levels in ischemic gastrocnemius on day 3 following surgery (n = 4). The data points are represented as mean ± SD. ^##^ *p* < 0.01, ^###^ *p* < 0.001 vs. sham; * *p* < 0.05, ** *p* < 0.01 vs. HLI.

The inflammatory response within the ischemic microenvironment is closely associated with angiogenesis. The flow cytometry analysis revealed that PF (20 mg/kg/d) reduced the proportions of neutrophils (CD45^+^CD11b^+^Ly6G^+^) and macrophages in ischemic gastrocnemius tissue on day 3 after surgery compared to the HLI group (Figure 6A–E). Additionally, the ELISA results showed higher concentrations of IL-1β, TNF-α, and IL-6 in serum samples of the HLI group on day 3 and 7 following surgery than in the sham group. However, PF administration suppressed this elevation (Figure 6F–K). Furthermore, PF conspicuously reduced mRNA levels of TNF-α and IL-6 in ischemic gastrocnemius tissue at 3 days post-surgery (Figure 6L–M). These results indicated that PF mitigates the acute inflammatory responses during vascular remodeling by decreasing the expression of inflammatory factors and inhibiting macrophage and neutrophil recruitment, which exerted positive effects on angiogenesis in ischemic tissue during its rehabilitation.

### 2.7. PF Regulated the Phenotypic Transformation of Macrophages In Vivo and In Vitro Assays

**Figure 7:**
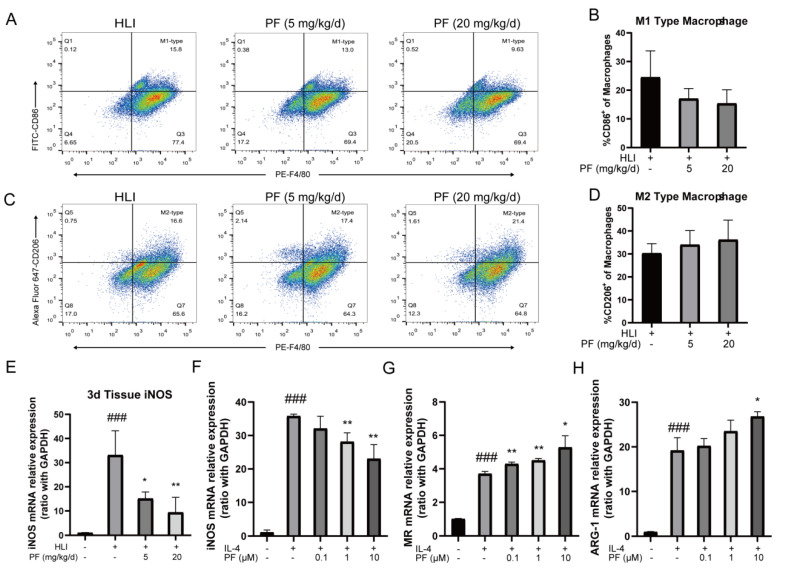
PF regulated the phenotypic transformation of macrophages in vivo and in vitro experiments. (**A**,**C**) Typical flow cytometry images of pro-inflammatory and anti-inflammatory macrophages in mice treated or not with PF (lower and high dose) on day 3 after surgery. Quantitative data were shown in (**B**,**D**) (n = 4–5). (**E**) Expression levels of iNOS mRNA were analyzed in ischemic gastrocnemius by an RT-PCR analysis on day 3 after surgery (n = 4). (**F**) Quantitative data of pro-inflammatory macrophages markers iNOS mRNA expression in BMDMs treated with DMSO or PF (0.1, 1, and 10 μM) for 24 h were evaluated using RT-PCR (n = 3). (**G**,**H**) Quantitative data of anti-inflammatory macrophages markers MR and ARG-1 mRNA expression in BMDMs exposed to DMSO or PF (0.1, 1, and 10 μM) for 24 h were evaluated using RT-PCR (n = 3). The data points are represented as mean ± SD. ^###^ *p* < 0.001 vs. sham; * *p* < 0.05, ** *p* < 0.01 vs. HLI.

**Figure 8:**
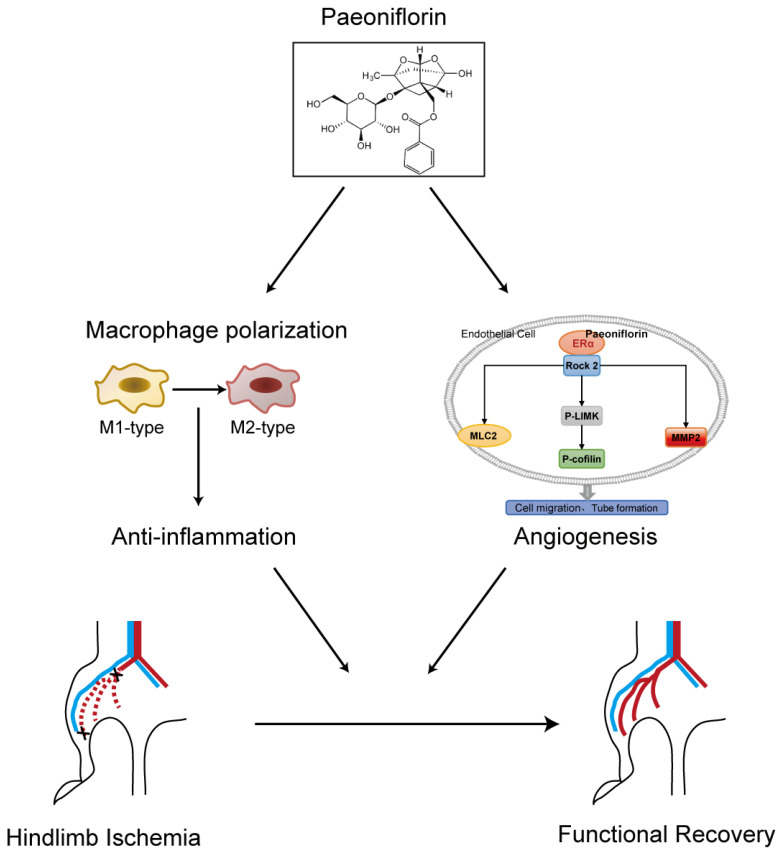
A concise graph illustrating the proposed angiogenesis mechanism of PF was presented.

Immunomodulation profoundly influences angiogenesis during the recovery of ischemic tissue injury. In vivo experiments employed flow cytometry to observe the proportions of pro-inflammatory macrophages and anti-inflammatory macrophages in gastrocnemius of the HLI mice. The findings indicated that compared to the HLI group, the proportion of pro-inflammatory macrophages (CD86^+^ macrophages) decreased in the PF-H group (20 mg/kg/d) at 3 days post-surgery, while that of anti-inflammatory macrophages (CD206^+^ macrophages) increased (Figure 7A–D). The RT-PCR results demonstrated that PF treatment suppressed the mRNA expression of iNOS in ischemic gastrocnemius tissue compared to the model group (Figure 7E). In vitro studies also showed that PF inhibited pro-inflammatory polarization by reducing iNOS mRNA expression in primary BMDMs induced by LPS/IL-4 (Figure 7F). Additionally, PF promoted anti-inflammatory polarization by enhancing the mRNA expression of MR, indicating PF’s ability to shift macrophages towards the anti-inflammatory phenotype in vitro (Figure 7G,H). These results indicated that PF regulates the phenotypic transformation of macrophages, mitigates acute inflammatory responses during vascular remodeling, and promotes the rehabilitation of ischemic tissue. The above results suggested that PF promoted the repair of ischemic tissue injury in an HLI mouse model through multiple mechanisms involving pro-angiogenesis by activating the ERα/ROCK-2 pathway and anti-inflammatory behavior by inducing macrophages polarizing toward the anti-inflammatory phenotype (Figure 8).

## 3. Discussion

Patients with peripheral artery disease (PAD) face severe limitations in daily activities due to hind limb ischemia, which can lead to amputation or death. Current treatments, including medication and non-surgical interventions, are insufficient for the needs of PAD patients. The tissue healing process after limb ischemia comprises three phases: (1) the necrosis and apoptosis of numerous large damaged cells; (2) the inflammation phase, characterized by the recruitment of neutrophils and macrophages to the site of the ischemic injury area; and (3) the angiogenesis phase and remodeling of the pre-existing vascular system, which is a constituent of tissue remodeling that involves the control of the extent of ischemic damage [26,27]. The inflammatory response is a key component of the ischemic micro-environment, which is intimately connected with angiogenesis [28,29]. Developing medications with both pro-angiogenic and anti-inflammatory effects has emerged as a new strategy. Numerous reports revealed that PF has the character of anti-inflammation [18]. PF, known for its anti-inflammatory properties, has also shown promise in accelerating wound healing and increasing capillary density in our previous studies. In this study, we found that PF improved the blood flow recovery, inhibited the apoptosis in the ischemic gastrocnemius, and facilitated revascularization in a mouse model of hindlimb ischemia.

Angiogenesis, a complex process, is determined with cytokines, growth factors, and chemokines. VEGF, a crucial regulator, enhances vascular endothelial cell proliferation, migration, and tube formation and inhibits apoptosis [30]. In ischemic tissue, ERα receptors are activated [31], and MMPs play a role in endothelial cell migration and proliferation by modulating VEGF expression [32]. MMPs are considered pro-angiogenic factors, directly involved in angiogenesis. Our experimental results confirm the interaction and reciprocal regulation of these proteins during angiogenesis. PF enhances endothelial cell activity, increases capillary density, fosters angiogenesis, and mitigates ischemic tissue injury, possibly by modulating these protein expressions.

In vitro, PF was found to enhance endothelial cell migration by activating the ERα/ROCK-2 pathway, thereby facilitating tube formation. Angiogenesis involves four main steps: (1) the degradation of the basement membrane by proteolytic enzymes like MMPs secreted by vascular endothelial cells; (2) endothelial cells migrating outward to generate capillary sprouts; (3) the formation of tube-like structures through endothelial cell proliferation, migration, adhesion, and connection; and (4) the establishment of new branch points and the interconnection of new tubes to construct a vascular network [33,34]. Endothelial cell proliferation and migration are crucial for angiogenesis. PF was observed to promote HUVEC proliferation and enhance stress fiber formation, leading to increased endothelial cell migration and tube formation. Molecular docking results suggested that PF can bind to ERα, activating it through non-genomic pathways to upregulate ROCK-2 expression on the cell membrane. ROCK-2, a downstream factor of GTPase RhoA, influences actin cytoskeleton and stress fiber formation [35,36,37]. ROCK also phosphorylates cofilin via Lim kinase, inhibiting actin depolymerization, increasing myosin light chain (MLC) phosphorylation, and enhancing stress fiber assembly, endothelial cell contractility, and migration. Additionally, ROCK activation can stimulate MMPs to degrade the basement membrane, promoting endothelial cell migration [38]. Therefore, PF likely promotes stress fiber generation and endothelial cell migration by activating ERα/ROCK-2 pathways, thereby enhancing endothelial cell tube-forming capability.

Moreover, our study revealed that PF attenuated neutrophil and macrophage recruitment, reduced inflammatory factor expression, regulated macrophage phenotypic transformation in vivo and in vitro, and mitigated severe inflammation during vascular remodeling, thereby facilitating post-ischemic tissue repair. The inflammatory phase following tissue ischemia is characterized by neutrophil infiltration followed by monocyte and macrophage recruitment [39]. Initially, pro-inflammatory macrophages drive the inflammatory response to clear cell debris and bacteria. Later in inflammation, anti-inflammatory macrophages accumulate to dampen inflammation, initiate angiogenesis, and promote tissue healing. Excessive pro-inflammatory macrophages have been implicated in exacerbating ischemic disease and delaying recovery in the late inflammatory phase [40,41,42,43]. Therefore, PF reduces inflammatory cell accumulation, suppresses inflammatory factor release, and promotes macrophage polarization towards the anti-inflammatory phenotype, offering a promising therapeutic approach for PAD. However, further investigation is warranted to clarify the exact mechanisms involved in this process.

Compared to traditional treatments, paeoniflorin (PF) offers a milder anticoagulant effect with reduced bleeding risk by inhibiting platelet activation and lowering blood viscosity [44,45,46]. It also enhances angiogenesis through a multi-target approach, modulating VEGF, PI3K/Akt, Nrf2, and HIF-1α pathways for healthier vascular regeneration [47,48]. Additionally, PF provides broad anti-inflammatory effects by regulating multiple signaling pathways while maintaining immune balance [18,49,50,51,52], resulting in fewer side effects than conventional anti-inflammatory drugs. Overall, PF demonstrates significant therapeutic potential in anticoagulation, angiogenesis, and inflammation management. Its multi-target mechanism and lower toxicity compared to traditional treatments may provide a distinct advantage in clinical applications.

Together, in this study, we demonstrated that PF improved blood perfusion and limb function in an HLI mouse model by promoting angiogenesis and enhancing ischemic tissue repair. Mechanistically, PF activated the ERα/ROCK-2 migration pathway, leading to increased stress fiber generation and endothelial cell migration while also promoting endothelial cell proliferation. Additionally, PF reduced inflammatory cell accumulation (neutrophils and macrophages) and regulated macrophage phenotypic transformation in vivo and in vitro, resulting in decreased inflammatory factor release and alleviation of severe inflammation during revascularization after ischemic injury, thus facilitating ischemic tissue rehabilitation. These findings unveil a novel role of PF in promoting angiogenesis and suppressing inflammation in the vascular remodeling process of ischemic limb lesions, providing insights into potential treatments for PAD. Moreover, PF’s low toxicity and safety profile make it a potential contender for drug development.

## 4. Materials and Methods

### 4.1. Reagents

Paeoniflorin (purity ≥ 98%) was provided by Chengdu Desite Bio-Technology Co., Ltd. (Chengdu, China), Avertin (T48402), simvastatin (sim) (S6196), LPS (L2630), collagenase II (C6885), and dispase II (D4693) were obtained from Sigma-Aldrich (St. Louis, MO, USA). PerCP anti-mouse CD45 (103129), Brilliant Violet 421 anti-mouse/human CD11b (101251), PE anti-mouse F4/80 (123110), PE anti-mouse Ly-6G (127607), FITC anti-mouse CD86 (105005), and Alexa Fluor 647 anti-mouse CD206 (141711) were acquired from BD Biosciences (San Jose, CA, USA). Antibodies of VEGFA (ab51745) and MMP9 (ab38898) were acquired from Abcam (Cambridge, MA, UK). Antibodies of ERα (SC-543) was acquired from Santa Cruz Biotechnology (Santa Cruz, TX, USA). Antibodies for Rho-associated coiled-coil kinase 2 (ROCK-2) (9029), MLC2 (3672), P-MLC2 (3674), LIMK1 (3842), P-LIMK1(3841), cofilin (5175), p-cofilin (3313) and glyceraldehyde-3-phosphate dehydrogenase (GAPDH) (2118S), and MMP2 (87809) were obtained from Cell Signaling Technology (Danvers, MA, USA). IL-1β (E-EL-M0037c), TNF-α (E-EL-M0049c), and IL-6 (E-EL-M0044c) mouse ELISA kits were purchased from Elabscience^®^ (Wuhan, China). Human umbilical vein endothelial cells (HUVECs, HUVEC-20001), endothelial cell growth medium (EGM), and fatal bovine serum (FBS) were sourced from Cyagen Biosciences Inc. (Guangzhou, China). Recombinant murine macrophage colony stimulating factor (M-CSF) (315-02) and recombinant murine IL-4 (214-14) were obtained from Pepro Tech (Rocky Hill, NJ, USA). TRIzol reagent (ET111), All-in-One First-Strand cDNA Synthesis Kit (AT341) and PerfectStart Green qPCR SuperMix (AQ602) were get from Transgen (Beijing, China).

### 4.2. Animals

Male C57BL/6 mice, 6–8 weeks old and weighing 20–24 g, were procured from SiPeiFu (Beijing, China) Biotechnology Co. Ltd. (Beijing, China) and housed at the Animal Center of Tianjin University of Traditional Chinese Medicine (Tianjin, China). The mice were kept in a controlled environment with a temperature of 22–25 °C and a 24 h light/dark cycle; they were fed standard laboratory chow and had unrestricted access to water. The animal care and experimental procedures involved were permitted by the Animal Ethics Committee of Tianjin University of Traditional Chinese Medicine and conducted in accordance with the approved Guidelines for Using the Laboratory Animals (Approval reference number: TCM-LAEC2019088).

### 4.3. Establishment of Hind Limb Ischemia (HLI) Model and Drug Treatment

HLI mice were induced according to our previous studies [53]. The proximal femoral artery, a branch of the internal iliac artery, was exposed by dissecting the inguinal fat pads from the peritoneal linings using micro-surgical forceps. After separating the femoral vein and artery from the surrounding connective tissue sheath, the upper and lower ends of the femoral artery were ligated, and the section of the artery between the ligatures was excised. Finally the skin incision was sutured layer by layer with 6-0 needles. The mice that underwent the surgeries were subdivided into five groups: sham + vehicle; HLI + vehicle; HLI + 10 mg/kg/d sim; HLI + 5 mg/kg/d PF; HLI + 20 mg/kg/d PF. Intragastric administration was given for the five groups of mice every day until they were harvested.

### 4.4. Laser Doppler Measurements

The laser Doppler perfusion imaging system (moor instruments, Axminster, Devon, UK) was utilized to measure the blood perfusion of the hindlimb of the mice from four experimental groups both before and after surgery as well as at 3, 7, 14, and 28 days post-femoral artery resection. The moorLDI laser Doppler imager review V 6.0 analysis software is utilized to analyze the blood flow ratio of the ischemic (right) to the control (left), thus revealing the recovery of blood velocity in the ischemic hind limbs.

### 4.5. Enzyme-Linked Immunosorbent (ELISA) and Serum Indices Assay

After anesthetizing the mice, we collected the peripheral blood from the retro-orbital vein and centrifuged the collected at 4000 rpm for 15 min (4 °C) to collect supernatant. Serum levels of IL-1β, IL-6, and TNF-α were measured by ELISA following the instructions of manufactures. A fully automatic biochemical analyzer (Vital Scientific, Netherlands) was used to detect the serum levels of CK, LDH, ALT, TP, ALB, T-BIL, AST, BUN, and CRE of all mice in each group.

### 4.6. Histological Analysis of Ischemic Limbs

We collected ischemic gastrocnemius at 28 days post-surgery and fixed the muscle tissues with 4% paraformaldehyde for 48 h. The samples were then dehydrated, embedded, and sectioned into 5 µm thick slices. Then, we stained sections with hematoxylin and eosin (H&E) for detecting muscle recovery from ischemic injury.

### 4.7. Terminal Deoxynucleotidyl Transferased UTP Nick End Labeling (TUNEL) Assay

The cellular apoptosis of the ischemic hindlimb muscles was observed at 7 days after surgery. The sections, after being deparaffinized and rehydrated, underwent treatment with 0.1% Triton X-100 for 8 min and were washed twice with PBS. Then, sections were incubated again in the reaction mixture for 1 h while maintaining an ambient temperature of 37 °C. Finally, we placed these sections under a fluorescence microscope and collected images.

### 4.8. Immunofluorescence

Mice were administered an intravenous injection of Griffonia (Bandeiraea) Simplicifolia Lectin I (50 µL/20 g) 30 min before being sacrificed. The sections were exposed to the primary antibodies of Griffonia (Bandeiraea) Simplicifolia Lectin I overnight at 4 °C and the negative control with the absence of primary antibodies. On the subsequent day, these sections underwent incubation in the dark with DyLight^®^594 ANTI-GOAT IgG (H+L) secondary antibody for a period of 20 min at room temperature. Finally, all sections were observed and photographed with Zeiss Laser Scanning Confocal Microscope (Jena, Germany).

### 4.9. HUVECs Cell Viability and Cell Proliferation Assays

HUVECs (passages 2–5) were maintained in EGM within an incubator set at 37 °C and 5% CO_2_. The CCK-8 detection kit was utilized to measure the viability of HUVECs, while the BrdU kit (Roche, Basel, Switzerland) was utilized to evaluate cell proliferation.

### 4.10. Transwell

Transwell culture plates (well diameter of 6.5 mm) with polycarbonate filters (bore diameter of 8 μm) were also utilized to measure chemotaxis and migration. The 24-well culture plates were all installed with cell chambers. Fibronectin was pre-coated on the upper and lower surfaces of the chambers. We prepared EGM culture medium in 24-well plates, seeded cells into the upper chamber cavities of each well, and then incubated the plates for 4 h at 37 °C, and finally, we stained the migrated cells with crystal violet.

### 4.11. Tube Formation Assay

The Matrigel was precooled in advance at 4 °C overnight, which was added into 96-well culture plates (50 μL/well), and cultured it at 37 °C for 1 h for its immobilization. Afterward, the cell suspensions were added into the culture plates for incubation of 18 h. Finally, the images were photographed with a Nikon microscope (Tokyo, Japan) and assessed with Adobe Photoshop CS5.

### 4.12. Western Blotting Assay

For the Western blotting assay, HUVECs and the frozen gastrocnemius were mixed in lysis buffer. Protein concentration was determined utilizing a BCA kit. Samples underwent separation via SDS-PAGE, subsequent transfer onto PVDF membranes, and then blocking with skimmed milk powder. The membranes were then coated at 4 °C for 12 h with primary antibodies targeting VEGFA, ERα, MMP9, MMP2, ROCK-2, p-cofilin, cofilin, p-MLC2, MLC2, p-LIMK1, LIMK1, and GAPDH. After being washed, the membranes were coated with the relevant secondary antibodies. Finally, the bands were visualized, and image analysis was performed utilizing ImageJ software (version 1.4.3.67). The expression of the target proteins was normalized to GAPDH to ensure accurate quantification.

### 4.13. Molecular Docking

For the molecular docking analysis, we downloaded the SDF file of the 2D structure of PF and imported it into Chem3D software (version 18.1) to verify its spatial structure. The 3D molecular structure of ERα (PDB ID: 5fqp) in PDB format was acquired from the PDB database (https://www.rcsb.org/ (accessed on 24 December 2023)). The protein structure was then imported into AutoDock Tools (version 1.5.6), where water molecules were removed, the protein was dehydrated, and non-polar hydrogens were added. AutoDock Tools Vina (version 1.5.6) was applied to proceed the docking analysis between the protein and the ligand. The optimal binding pose was chosen based on the docking conformation exhibiting the highest binding score. The visualization and analysis of the docking results were conducted using PyMOL 4.5.0.

### 4.14. Flow Cytometry Analysis

The ischemic gastrocnemius of HLI mice was collected at 3 days after surgery and was digested with the balanced salt solution of Hanks that contains 9 mg of dispase II and 7.2 mg of collagenase II at 37 °C for 1.5 h. The cells were stained for 30 min with PerCP anti-mouse CD45, BV421 anti-mouse CD11b, PE anti-mouse F4/80, PE anti-mouse Ly-6G, FITC anti-mouse CD86, and Alexa Fluor 647 anti-mouse CD206. The stained single cells were then fixed with a 1% cell fixative solution and sorted using the BD FACS Aria III flow cytometer (San Jose, CA, USA).

### 4.15. Primary Bone Marrow Derived Macrophages (BMDMs) Culture

To obtain bone marrow cells, intact femurs and tibias were aseptically harvested from the hindlimb of C56BL/6 mice. The collected cells were nurtured in RPMI 1640 medium with 20 ng/mL recombinant murine M-CSF for 7 days to induce differentiation into BMDMs. Then, cells were processed with 20 ng/mL recombinant murine IL-4 and 100 ng/mL LPS for 24 h. The mRNA levels of the activated markers of pro-inflammatory macrophages and anti-inflammatory macrophages were determined using quantitative reverse transcriptase polymerase chain reaction (RT-PCR).

### 4.16. Quantitative RT-PCR Assay

Total RNA was isolated from ischemic hindlimb muscle tissues of the hindlimb and BMDMs through the utilization of tissue homogenizers in conjunction with TRIzol reagent. The RNA was reverse transcribed into cDNA using the All-in-One First-Strand cDNA Synthesis Kit. The mRNA levels were measured by employing PerfectStart Green qPCR SuperMix in QuantStudio 6 Flex (Thermo Fisher Scientific, Waltham, MA, USA). Glyceraldehyde-3-phosphate dehydrogenase (GAPDH) served as control for normalization. The relative expression levels of genes were assessed using the 2^−ΔΔCt^ quantification method. The primers utilized for the mouse samples were as follows (Table 1):

### 4.17. Statistical Analysis

Statistical analysis was conducted using GraphPad Prism 8. Data were presented as mean ± SD. Differences between two groups were evaluated using Student’s *t*-test.

## Figures and Tables

**Table 1:** Primer sequences.

Gene	Primer Forward	Primer Reverse
*GAPDH*	GGTGCTGAGTATGTCGTGGA	CCTTCCACAATGCCAAAGTT
*iNOS*	GTTCTCTGGGAAATCGTGGA	GGAAATTGGGGTAGGAAGGA
*TNF-α*	GAAGAGAACCTGGGAGTAGATAAGG	GTCGTAGCAAACCACCAAGC
*IL-6*	GTTCTCTGGGAAATCGTGGA	GGAAATTGGGGTAGGAAGGA
*MR*	CAAGGAAGGTTGGCATTTGT	CCTTTCAGTCCTTTGCAAGC
*Arg-1*	TGAGAGACCACGGGGACCTG	GCACCACACTGACTCTTCCATTC

## Data Availability

The original contributions presented in this study are included in the article/supplementary material. Further inquiries can be directed to the corresponding authors.

## Supplementary Materials

The following supporting information can be downloaded at: https://www.mdpi.com/article/10.3390/ph18020272/s1, Figure S1: PF had no side effects in a mouse model of HLI. Figure S2. PF effected tubule-related genes on mRNA expression in HUVECs.
