# Supplementary material for: Paeoniflorin Attenuates Limb Ischemia by Promoting Angiogenesis Through ERα/ROCK-2 Pathway
Source: Pharmaceuticals (Basel). 2025 Feb 19;18(2):272. doi: 10.3390/ph18020272 (PMC11859641; doi:10.3390/ph18020272)
Supplement: Supplementary file 1 [file pharmaceuticals-18-00272-s001.zip › pharmaceuticals-3461171-supplementary.pdf]

Supplementary Figures

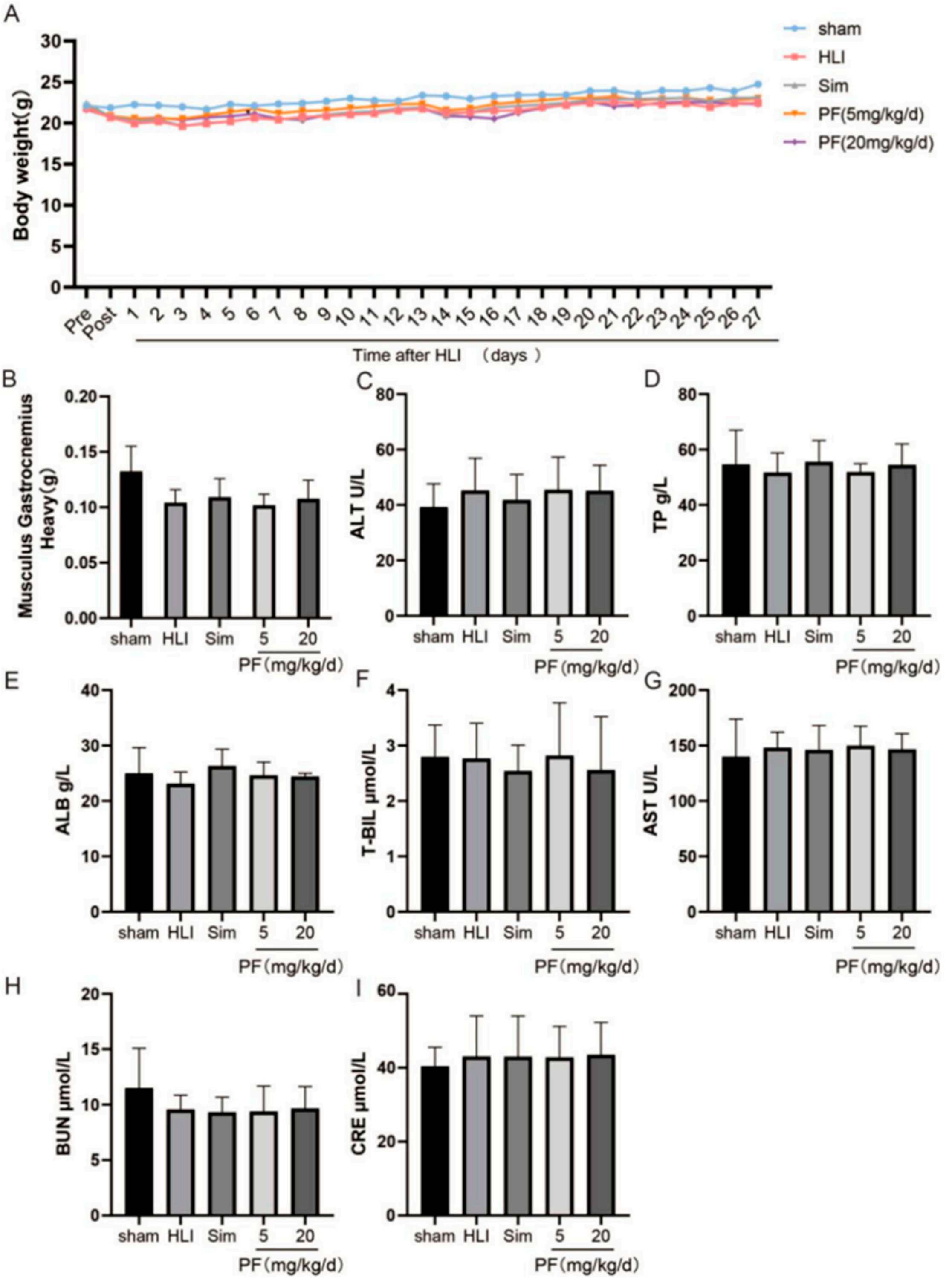

Figure S1. PF had no side effects in a mouse model of HLI. (A) Mouse body weight was detected every day after surgery (n = 5-6). **(B)** Gastrocnemius heavy were detected on day 28 after surgery (n = 5-6). **(C-I)** The content of ALT, TP, ALB, T-BIL, AST, BUN and CRE in serum of

mice on 28 days were detected(n = 5). The data points are represented as mean ± SD.

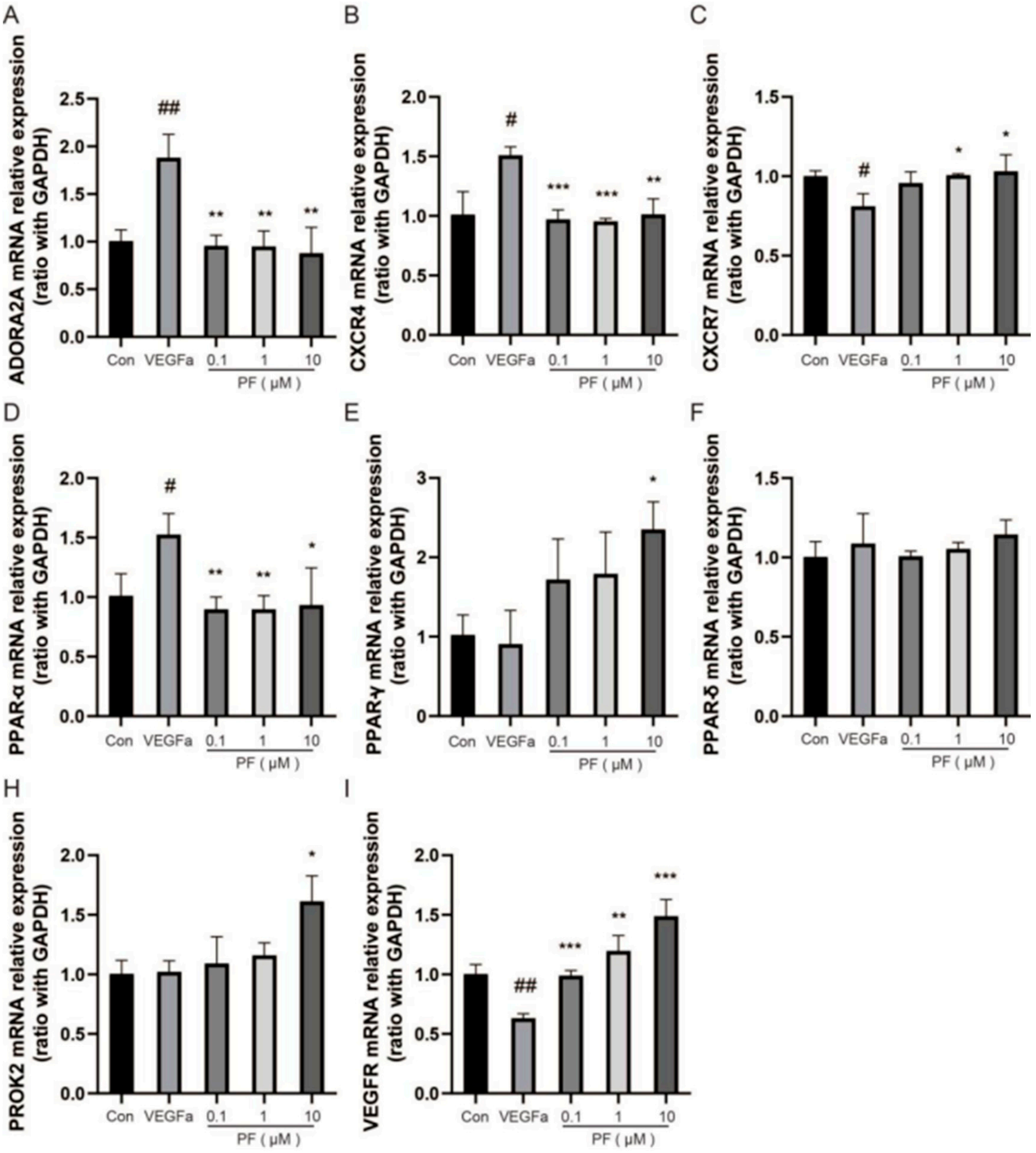

Figure S2. PF effected tubule-related genes on mRNA expression in HUVECs. (A-I) Quantitative data of ADORA2A, CXCR4, CXCR7, PPARA, PPARD, PPARG, PROK2 and VEGFR mRNA expression in HUVECs treated with DMSO or PF (0.1, 1 and 10 μM) for 24 h were analyzed by RT-PCR (n = 3). VEGF is a positive control. The data points are represented as mean ± SD. $^{*}P < 0.05$, $^{**}P < 0.01$ *vs*. Control.
